# Cardiac Fibroblasts and Myocardial Regeneration

**DOI:** 10.3389/fbioe.2021.599928

**Published:** 2021-03-25

**Authors:** Wangping Chen, Weihua Bian, Yang Zhou, Jianyi Zhang

**Affiliations:** ^1^Department of Biomedical Engineering, School of Medicine and School of Engineering, University of Alabama at Birmingham, Birmingham, AL, United States; ^2^Department of Cardiovascular Surgery, The Second Xiangya Hospital, Central South University, Changsha, China

**Keywords:** cardiac fibroblast, myocardial infarction, extracellular matrix, stem cells, reprogramming

## Abstract

The billions of cardiomyocytes lost to acute myocardial infarction (MI) cannot be replaced by the limited regenerative capacity of adult mammalian hearts, and despite decades of research, there are still no clinically effective therapies for remuscularizing and restoring damaged myocardial tissue. Although the majority of the cardiac mass is composed of cardiomyocytes, cardiac fibroblasts (CFs) are one type of most numerous cells in the heart and the primary drivers of fibrosis, which prevents ventricular rupture immediately after MI but the fibrotic scar expansion and LV dilatation can eventually lead to heart failure. However, embryonic CFs produce cytokines that can activate proliferation in cultured cardiomyocytes, and the structural proteins produced by CFs may regulate cardiomyocyte cell-cycle activity by modulating the stiffness of the extracellular matrix (ECM). CFs can also be used to generate induced-pluripotent stem cells and induced cardiac progenitor cells, both of which can differentiate into cardiomyocytes and vascular cells, but cardiomyocytes appear to be more readily differentiated from iPSCs that have been reprogrammed from CFs than from other cell types. Furthermore, the results from recent studies suggest that cultured CFs, as well as the CFs present in infarcted hearts, can be reprogrammed directly into cardiomyocytes. This finding is very exciting as should we be able to successfully increase the efficiency of this reprogramming, we could remuscularize the injured ventricle and restore the LV function without need the transplantation of cells or cell products. This review summarizes the role of CFs in the innate response to MI and how their phenotypic plasticity and involvement in ECM production might be manipulated to improve cardiac performance in injured hearts.

## Introduction

The limited regenerative ability of adult mammalian hearts (Porrello et al., 2011) cannot replace the millions of cardiomyocytes that are lost to myocardial infarction (MI) (Prabhu and Frangogiannis, 2016). Instead, the damaged tissue is remodeled and replaced by non-contractile scar tissue, which impedes cardiac function and can eventually lead to catastrophic heart failure (HF) (Wang and Guan, 2010). HF is among the leading causes of hospitalization and death worldwide (Cahill et al., 2017) and is likely to become even more prevalent in response to lifestyle changes and the overall aging of the population. Currently, the available treatment options are generally limited to pharmacological therapies and surgical interventions such as stents and coronary artery bypass graft surgery, which can delay disease progression but fail to increase the number of functional cardiomyocytes and, consequently, do not address the root cause of the decline in cardiac performance (Lin and Pu, 2014). Thus, the development of novel strategies for replacing the myocardial scar with active contractile tissue is perhaps the fundamental goal of cardiovascular research (Carvalho and de Carvalho, 2010).

Although the majority of the cardiac mass is composed of cardiomyocytes (Zhou and Pu, 2016), cardiac fibroblasts (CFs) are one type of the most numerous cells in the heart (Sadoshima and Weiss, 2014). Their exact proportion varies depending on species and age, and measurements can also be influenced by the techniques and marker(s) used for identification (Zak, 1974; Nag, 1980; Banerjee et al., 2007), because CFs can assume a variety of phenotypes and descend from numerous developmental origins (Sadoshima and Weiss, 2014). Nevertheless, CFs can be broadly defined as mesenchymal cells that reside in the cardiac interstitium (Souders et al., 2009), and they are the primary drivers of remodeling in response to both physiological and pathological conditions (Sadoshima and Weiss, 2014). Thus, they serve a critical role in the immediate aftermath of MI by producing the scar tissue required to maintain the structural integrity of the chamber walls and prevent rupture, but the scar also impedes contractile performance, disrupts electromechanical coupling (which can generate arrhythmias), and induces mechanical stress that can lead to additional cardiomyocyte toxicity and infarct expansion.

**FIGURE 1:**
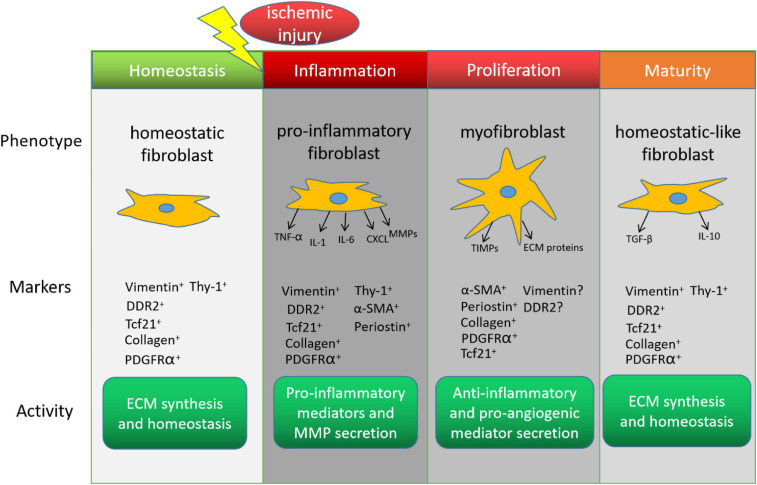
Cardiac fibroblasts (CFs) in the endogenous response to myocardial infarction. The phenotype, marker expression, and activity of CFs change during recovery from myocardial infarction. DDR2, discoidin domain receptor tyrosine kinase 2; Tcf21, transcription factor 21; PDGFRα, platelet-derived growth factor receptor α; α-SMA, α smooth-muscle actin; TNF-α, tumor necrosis factor α; IL, interleukin; CXCL, C-X-C motif ligand; MMP, matrix metalloproteinase; ECM, extracellular matrix; TIMP, tissue inhibitors of metalloproteinase; TGF-β, transforming growth factor β.

The healing process after MI can be divided into three distinct but overlapping stages–inflammation, proliferation, and maturity–and each stage is associated with a different CF phenotype (Frangogiannis, 2006; Figure 1). The inflammatory phenotype is characterized by the secretion of cytokines and chemotactic factors that promote the infiltration of neutrophils and monocytes (Sandanger et al., 2013; Shinde and Frangogiannis, 2014), which clear cellular debris, and by the production of matrix metalloproteinases (MMPs), which initiate remodeling by degrading the existing extracellular matrix (ECM). In the proliferative stage, CFs transform into myofibroblasts, which express the contractile protein α-smooth muscle actin (α-SMA), vigorously proliferate, and become the dominant effector molecules of the repair process by secreting both anti-inflammatory and pro-angiogenic molecules, and by generating the new ECM (Shinde and Frangogiannis, 2014; Ma et al., 2017). Unlike fibroblasts, myofibroblasts are specialized cells that possess a more contractile and synthetic phenotype than fibroblasts. The lineage tracing of Periostin+ myofibroblasts did not show expression of the endothelial cell marker CD31 after MI (Kanisicak et al., 2016), suggesting the limited plasticity of myofibroblasts to transdifferentiate to other cardiac cell. CFs continue to produce anti-inflammatory cytokines (e.g., interleukin 10) and pro-fibrotic factors (e.g., transforming growth factor β1) during the early maturation stage (Chen and Frangogiannis, 2013), and then the number of myofibroblasts declines as the cells transition to a phenotype that promotes scar maturation and maintains homeostasis in the remodeled myocardium (Ma et al., 2017). More recently, Fu et al. (2018) dissected the dynamic states of CFs during post-myocardial infarction remodeling by lineage tracing of cells expressing *Tcf21*, *Postn* and *Acta2* genes. Consistently, they identified proliferating activated fibroblasts early at post-MI day 2–4 and the α-SMA+ myofibroblasts at day 3–7 after injury. They also discovered a new differentiated state of fibroblasts in the mature scar beyond 10 days after injury. These CFs, termed as matrifibrocytes, highly express bone and cartilage-related ECM genes like *Chad*, *Cilp2* and *Comp*, which are common gene signatures of chondrocytes and osteoblasts, making CFs more specialized to support mature scar. Importantly, deletion of these cells in the scar impaired the cardiac function, suggesting an indispensable role of matrifibrocytes in the homeostasis of scarred hearts.

**FIGURE 2:**
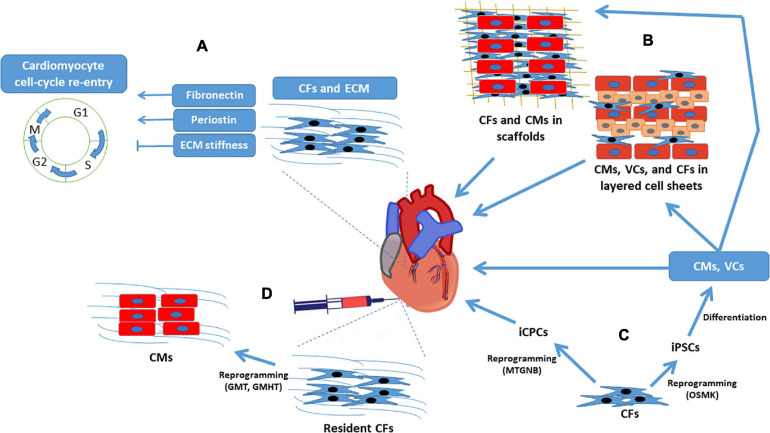
Cardiac fibroblasts for regenerative myocardial therapy. Clockwise from top left: **(A)** CFs secrete cytokines that directly regulate cardiomyocyte cell-cycle activity, and the structural proteins produced by CFs influence cardiomyocyte proliferation by modulating ECM stiffness. **(B)** CFs have a supportive role in engineered myocardial tissues and **(C)** can be reprogrammed into iPSCs or iCPCs; iPSCs are differentiated into cardiomyocytes, endothelial cells, and smooth-muscle cells before administration to infarcted hearts or assembly into engineered myocardial tissues, whereas iCPCs spontaneously differentiate into cardiac cells after delivery to the heart. **(D)** CFs can also be reprogrammed directly into cardiomyocyte-like cells both *in-vitro* and *in-vivo*. CF, cardiac fibroblast; CM, cardiomyocyte; ECM, extracellular matrix; GHMT, Gata4, Hand, Mef2c, Tbx5; GMT, Gata4, Mef2c, Tbx5; iCPC, induced cardiac progenitor cell; iPSC, induced pluripotent stem cell; MTGNB, Mesp1, Tbx5, Gata4, Nkx2.5, Baf60c; OSMK, Oct4, Sox2, c-Myc, Klf4; VC, vascular cell.

While both the immediate benefits and long-term, often detrimental, effects of CFs on cardiac performance after an infarct event are well established, efforts to improve myocardial recovery via CF-based therapies have also become a prominent field of research. The remainder of this review focuses on how the role of CFs in ECM production might be exploited to limit infarct size and manipulate cardiomyocyte activity and proliferation, as well as the reprogramming of CFs into pluripotent cells or, perhaps, directly into cardiomyocytes (Figure 2).

## CF-Derived Extracellular Matrix in Myocardial Repair

Cardiac fibroblasts produce growth factors and other signaling molecules that directly regulate cardiomyocyte function (Torre-Amione et al., 1996; Long, 2001; Baudino et al., 2008), while also controlling the synthesis and degradation of the ECM (Baxter et al., 2008; Snider et al., 2009; Souders et al., 2009). The cardiac ECM was once believed to function primarily as an inert scaffold but is now known to be an ever-changing and plastic microenvironment that has a vital role in cardiac function and regeneration. The mammalian cardiac ECM consists of structural components such as collagens, fibronectin, tenascin, elastin, laminins, proteoglycans, and glycosaminoglycans (Lockhart et al., 2011), as well as dynamic, non-structural (i.e., matricellular) proteins that participate in critical signal transduction pathways (Rienks et al., 2014; Frangogiannis, 2017), some of which appear to regulate cardiomyocyte proliferation. Embryonic CFs produce fibronectin and heparin-binding EGF-like growth factor, which activate proliferation in cultured mouse cardiomyocytes via β1-integrin signaling (Ieda et al., 2009), while neonatal rat ventricular cells were significantly more proliferative when cultured with fetal cardiac ECM than with either neonatal or adult cardiac ECM, and the increase corresponded with 6- to 7-fold higher measures of fibronectin and periostin (Williams et al., 2014). Periostin is secreted by CFs after MI or pressure overload injury (Shimazaki et al., 2008) and has been shown to improve infarct size and cardiac function in infarcted rat hearts by activating integrin- and phosphatidylinositol-3 kinase (PI3K) signaling, which subsequently induces cardiomyocyte cell-cycle re-entry (Kuhn et al., 2007), while periostin deficiencies inhibited myocardial regeneration after MI in neonatal mice (Chen et al., 2017).

The structural ECM components produced by CFs can also modulate cardiac regeneration by altering ECM stiffness (Yahalom-Ronen et al., 2015). Stiffness of the ECM increases with the deposition and crosslinking of collagen, elastin, and laminin (Notari et al., 2018; Frangogiannis, 2019), and ECM stiffness and maturation induce cell-cycle arrest in neonatal rat and mouse cardiomyocytes, whereas more compliant ECM (i.e., ECM that can accommodate greater blood volume with smaller increases in pressure) promotes cardiomyocyte proliferation and cytokinesis (Yahalom-Ronen et al., 2015). Furthermore, the regenerative capacity of neonatal mouse hearts in response to apical resection is greatest on the first day after birth (P1) and declines rapidly thereafter (Porrello et al., 2011; Lam and Sadek, 2018; Notari et al., 2018), and analyses of the transcriptomes of P1 and P2 mice identified significant differences in the expression of ECM and cytoskeletal genes that contribute to ECM stiffness. Notably, cardiac regenerative capacity can be restored in mice by pharmacologically reducing stiffness on P3 (Notari et al., 2018), and the composition and stiffness of the ECM may also influence the myogenic differentiation of stem and bone-marrow–derived cells (Engler et al., 2006; Zhang et al., 2009; Wang et al., 2010; Hastings et al., 2015). Increases in cardiomyocyte cell-cycle activity have also been observed in patients after implantation of a left-ventricular assist device, which can mimic increases in myocardial compliance by reducing the hemodynamic load (i.e., mechanical unloading) (Canseco et al., 2015).

The role of the ECM in cardiac function and cardiomyocyte cell-cycle activity also has important implications for the use of CFs in cardiac tissue engineering, particularly for the development of thicker and vascularized constructs (Shimizu et al., 2006; Novosel et al., 2011; Haraguchi et al., 2012). Both the functional and biochemical properties of engineered cardiac tissues composed of CFs, cardiomyocytes, and a biorubber scaffold improved when the two cell populations were added sequentially (CFs first, then cardiomyocytes) rather than simultaneously (Radisic et al., 2006), and the structural support provided by dermal fibroblasts improved cell-cell interactions and the synchronous beating of cardiomyocytes in injectable beating mini heart tissues (Guerzoni et al., 2019). CFs have also been combined with cardiomyocytes and endothelial cells to produce native-like three-dimensional (3D) cardiac tissue with oriented structures and a vascular network, which is crucial for cell survival after transplantation (Tsukamoto et al., 2020), and the ECM produced by CFs has been used as a transfer medium to improve the retention of transplanted mesenchymal stem cells in ischemic myocardium (Schmuck et al., 2014).

## CFs as a Source of Induced-Pluripotent Cells

Because CFs are available in large numbers and phenotypically plastic, they are particularly useful for cell therapy and tissue engineering. The most common strategies involve reprogramming the cells into either induced pluripotent stem cells (iPSCs) or cardiac progenitor cells (iCPCs). iPSCs have an unlimited capacity for self-renewal and can differentiate into cells of any lineage, but have also been associated with tumorigenesis; thus, they are typically differentiated into cardiomyocytes and other cardiac-lineage cells before administration to infarcted hearts or assembly into engineered myocardial tissues. iPSCs were first generated via the overexpression of four transcription factors (Oct4, Sox2, Klf4, and c-Myc) in mouse and human dermal fibroblasts (Takahashi and Yamanaka, 2006; Takahashi et al., 2007), and have also been generated from endothelial cells (Lagarkova et al., 2010), hair-follicle cells (Illing et al., 2013), keratinocytes (Aasen et al., 2008) and peripheral blood cells (Okita et al., 2013). However, cardiomyocytes appear to be more readily differentiated from iPSCs that have been reprogrammed from human CFs (hciPSCs) than from other cell types: the purity of hciPSC-derived cardiomyocytes (hciPSC-CMs) exceeded 92% without any subsequent selection procedures, whereas iPSCs reprogrammed from human dermal FBs (hdiPSCs) or blood mononuclear cells (h_*BMC*_iPSCs) yielded cardiomyocyte populations that were only 60–85% pure (Ye et al., 2013; Zhang et al., 2015). This difference in differentiation efficiency can likely be attributed to the presence of epigenetic factors that the iPSCs retained from their tissues of origin (Kim et al., 2010; Noguchi et al., 2018; Toubiana et al., 2019). Furthermore, the Ca^2+^-handling profile of the hciPSC-CMs was more cardiac-like than the profiles of cardiomyocytes differentiated from hdiPSCs or h_*BMC*_iPSCs, and when sheets of hciPSC-CMs were transplanted into infarcted mouse hearts, the rate of engraftment was exceptionally high (>30%) 28 days later; the treatment was also associated with significant improvements in cardiac contractile function. It is also interesting to notice that, as an unwanted limit of current cardiac differentiation protocol, non-cardiomyocytes cardiac cells (NMCCs) also emerge during iPSC differentiation and easily to be transdifferentiated into myofibroblast cells. We found that the inhibition of myofibroblast cells derived from NMCCs could improve the efficacy of cardiac cell therapy (Gao et al., 2018).

Cardiac progenitor cells (iCPCs) are also highly proliferative and multipotent, but their intrinsic capacity for differentiation is restricted to the cardiac mesoderm lineage (Masino et al., 2004), and they can be delivered directly to the heart. Mouse CFs were first stably reprogrammed into iCPCs via the overexpression of five (Mesp1, Tbx5, Gata4, Nkx2.5, and Baf60c), or as many as eleven (Mesp1, Mesp2, Gata4, Gata6, Baf60c, SRF, Isl1, Nkx2.5, Irx4, Tbx5, and Tbx20), cardiac transcription factors (Lalit et al., 2016), and the yield of iCPCs was further increased by including 6-bromoindirubin-30-oxime (a canonical Wnt activator) and leukemia inhibitory factor (LIF; a JAK/STAT activator) during the reprogramming process. Both *in-vitro* and *in-vivo* studies confirmed that the iCPCs could differentiate into cardiomyocytes, endothelial cells (ECs), and smooth-muscle cells (SMCs), and iCPC transplantation significantly improved cardiac function and survival in a mouse MI model with no evidence of tumor formation. iCPCs also repopulated ECM scaffolds and differentiated into cardiomyocytes, ECs, and SMCs when injected through an aortic cannula into decellularized whole mouse hearts (Alexanian et al., 2020). Thus, both iPSCs and iCPCs dedifferentiated from CFs may provide a readily available, safe, and scalable source of contractile and vascular cells for regenerative myocardial therapies.

## Reprogramming CFs Into Induced Cardiomyocytes

Cardiac fibroblasts can also be directly reprogrammed into cardiomyocyte-like cells [induced cardiomyocytes (iCMs)] without first passing through an intermediate iPSC stage; however, the technique is quite new, so the optimal reprogramming protocol has yet to be identified and may vary depending upon how the cells will be used after reprogramming. iCMs were first generated in 2010 via the overexpression of three developmental transcription factors (Gata4, Mef2c, and Tbx5; i.e., the GMT protocol) in CFs: the reprogrammed cells expressed cardiomyocyte-specific markers, contracted spontaneously, and displayed cardiomyocyte-like electrophysiological properties and global gene-expression profiles (Ieda et al., 2010). Subsequent work demonstrated that the efficiency of the GMT protocol could be improved significantly by combining relatively high levels of Mef2c with lower levels of Gata4, and Tbx5 (Wang et al., 2015), or by adding Hand2 to the list of transduced genes (the GHMT protocol) (Song et al., 2012), and the activity of a transgenic, cardiomyocyte-specific troponin T promoter-reporter construct was more prevalent when iCMs were generated from mouse CFs by adding Nkx2.5 to the GHMT reprogramming protocol than via any other published combination of transcription factors (Addis et al., 2013). The expression of mature cardiomyocyte markers also varied depending on the relative proportions of Gata4, Mef2c, and Tbx5 (Wang et al., 2015), while the overall profile of cardiac-gene expression could be broadened by using a reprogramming cocktail composed of Tbx5, Mef2c, and Myocd (Protze et al., 2012).

Researchers are also investigating the mechanisms and signaling pathways that contribute to CF-iCM reprogramming by including small molecules in the established protocols. Adding Akt1 (protein kinase B) to the GHMT protocol dramatically increased spontaneous beating in reprogrammed iCMs and produced cells that were polynucleated, hypertrophic, and responsive to β-adrenoreceptor modulation, which suggests a more mature cardiomyocyte phenotype; furthermore, the role of Akt in iCM reprogramming appeared to be regulated upstream by insulin-like growth factor 1 (IGF1) and PI3K and facilitated downstream by target of rapamycin complex 1 (mTORC1) and forkhead box o3a (Foxo3a) (Zhou H. et al., 2015). A83-01, a selective inhibitor of TGF-β signaling, also increased spontaneous beating and the expression of cardiac genes such as *Actc1*, *Myh6*, and *Ryr2*, in GHMT-reprogrammed iCMs (Zhao Y. et al., 2015), which suggests that pro-fibrotic signaling pathways impede CF-iCM reprogramming, and the efficiency of GMT-reprogramming increased when Bmi1 activity was inhibited with shRNA during an early stage of the protocol (Zhou et al., 2016), which confirms that epigenetic factors could be key obstacles to CF-iCM reprogramming. MicroRNAs can also contribute to CF-iCM reprogramming, as evidenced by reports that pairing miR-133 overexpression with the GMT protocol significantly increased functional iCM yield by suppressing Snai1 (Muraoka et al., 2014), and that even in the absence of transcription factors, a combination of miR-1, miR-133, miR-208, and miR-499 induced mouse CFs to express cardiomyocyte-specific genes, beat spontaneously, and display cardiomyocyte-like organization of the sarcomere (Jayawardena et al., 2012).

## *In-situ* iCM Reprogramming

When lentiviruses encoding the same set of four microRNAs (miR-1, -133, -208, and -499) were administered directly to the hearts of mice after MI, the treatment appeared to reprogram resident CFs into iCMs and was associated with increases in ejection fraction and lower measures of fibrosis (Jayawardena et al., 2015), which suggests that the vast pool of CFs could serve as an endogenous source of new cardiomyocytes for regenerative therapy. Genetic lineage-tracing studies have shown that GMT retroviruses also reprogram resident mouse CFs into iCMs when injected immediately after coronary artery ligation: the reprogrammed CFs formed sarcomeres, displayed a cardiomyocyte-like gene expression profile, and were bi-nuclear and electrically coupled to endogenous cardiomyocytes, and the treatment was associated with significant improvements in measures of cardiac ejection fraction, stroke volume, and infarct size (Qian et al., 2012). Retroviral delivery of GHMT also converted resident CFs into iCMs and improved recovery from myocardial injury in mice–measures of ejection fraction increased 2-fold while infarct sizes declined by 50%–and the efficiency of CF-to-iCM reprogramming was greater than in animals treated with GMT alone (Song et al., 2012). *In-situ* GMT reprogramming of CFs into iCMs has also been performed with Sendai virus (Miyamoto et al., 2018) and adenovirus (Mathison et al., 2017), which are more suitable than lenti- or retroviruses for clinical applications, because the vectors are not integrated into the host genome and are unlikely to cause insertional mutagenesis; both approaches significantly improved recovery from myocardial injury in rodents, and reprogramming efficiency was greater when performed with the Sendai virus than with integrating retroviruses.

Observations from at least two studies (Qian et al., 2012; Song et al., 2012) suggest that the efficiency of iCM reprogramming, as well as the maturity of the reprogrammed cells, is greater when performed *in-situ* after MI than in culture, which suggests that properties of the infarcted heart can enhance iCM reprogramming. Whether the inflammatory signaling from neutrophils and macrophages (Prabhu and Frangogiannis, 2016) contribute to this enhancement has yet to be determined, and the results from studies of inflammation in direct reprogramming have been somewhat contradictory: the anti-inflammatory drug diclofenac promoted cardiac reprogramming in postnatal and adult fibroblasts (Muraoka et al., 2019), whereas shRNA-mediated knockdown of the pro-inflammatory regulators TLR3, NFKB1, and COX2 impeded the cardiac reprogramming of human fibroblasts (Zhou et al., 2019). Necrotic cardiomyocytes also release damage signals [i.e., damage-associated molecular patterns (DAMPs)] that activate CFs after MI, and measures of cardiac function and scar size in infarcted mouse hearts were significantly better after co-treatment with GMT and thymosin β4, which activates fibroblasts and promotes angiogenesis, than after treatment with GMT alone (Qian et al., 2012). Additional clues about how the environment of the infarcted myocardium may influence iCM reprogramming can be inferred from observations in cultured cells: the conversion rate of iCMs improved, and was accompanied by increases in MMP3 expression, when the cells were suspended in a 3D hydrogel that mimicked cardiac ECM (Li et al., 2016), and both the quantity and maturity of iCMs increased when reprogramming was conducted on microgrooved substrate (Sia et al., 2016). Mechanical properties of the damaged myocardium could also contribute to iCM reprogramming, because the stiffness of the scarred region likely changes in response to collagen deposition (Voorhees et al., 2015), and the maturation of iPSC-CMs can be improved by manipulating the stiffness of the culture substrate (Ribeiro et al., 2015).

## Conclusion and Perspectives

Cardiac fibroblast are key players in every stage of recovery from myocardial injury, and their roles in both beneficial and maladaptive fibrosis have been well established; however, more recent investigations have begun to evaluate whether their phenotypic plasticity and involvement in ECM production can be manipulated to promote myocardial regeneration. CFs could be targeted directly to promote the proliferation of endogenous cardiomyocytes by upregulating the production of matricellular ECM proteins that activate the cardiomyocyte cell-cycle or by altering structural components to reduce ECM stiffness. CFs are one type of most numerous cells in the heart, and the epigenetic profile of iPSCs generated from CFs, rather than other cell types, appears to be more favorable for differentiation into cardiac-lineage cells, which suggests that CFs could be an abundant source of cardiomyocytes for cell-based therapy and tissue engineering. iCPCs reprogrammed from CFs can differentiate into cardiomyocytes, ECs, and SMCs after transplantation into infarcted hearts and have been associated with improvements in cardiac function and survival with no evidence of tumorigenesis, while a number of protocols have been developed for reprogramming CFs directly into iCMs both *in vitro* and *in situ*, which could enable a promising therapeutic strategy to repopulate the myocardial scar with cardiomyocytes while avoiding the need for transplanted cells. However, the optimal combination of transcription factors and/or microRNAs for CF-iCM reprogramming has yet to be identified, and ongoing investigations into potential tumorigenicity of iPSC-CMs and the mechanisms that regulate both the generation of iCPCs and their differentiation into functional cardiac cells are required to facilitate the translation of these technologies to the clinic.

Certainly, challenges remain in our understanding of CF’s functions and plasticity as well as how the knowledge can be utilized to achieve heart regeneration. Although increasing evidence has shown that CFs and ECM significantly contribute to homeostasis and recovery after injury, the complexity of their crosstalk with cardiomyocytes and other cells remains largely unknown. Additionally, it is still unclear how the *in vivo* environment with changed ECM compositions influences fibroblast plasticity and integration of transplanted cardiomyocytes. It is also interesting to investigate whether *in situ* cardiac reprogramming will affect CF dynamics and ECM production, which might lead to synergetic benefit for heart tissue repair. Finally, most of the current findings are mainly from mouse studies. It is necessary and important to expand our understanding of cardiac fibroblasts in terms of their characteristics, behaviors, and functions in large animals and humans.

## Author Contributions

WC and JZ wrote the manuscript. JZ, YZ, and WB made the revision. JZ supervised the entire manuscript preparation process and figures design and creation. All authors approved the submission and publication of the manuscript.

## Conflict of Interest

The authors declare that the research was conducted in the absence of any commercial or financial relationships that could be construed as a potential conflict of interest.
